# Examining the recent trends in adolescent sexual and reproductive health in five countries of sub‐Saharan Africa based on PMA and DHS household surveys

**DOI:** 10.1186/s12978-021-01111-0

**Published:** 2021-06-17

**Authors:** Rornald Muhumuza Kananura, Peter Waiswa, Dessalegn Y. Melesse, Cheikh Faye, Ties Boerma

**Affiliations:** 1 Department of Health Policy Planning and Management, Makerere University School of Public Health, Mulago New-Complex, Kampala, Uganda; 2 Department of International Development, London School of Economics and Political Science, London, UK; 3 Makerere University Centre of Excellence for Maternal, Newborn and Child Health, Mulago New-Complex, Kampala, Uganda; 4 Global Health Department of Public Health Sciences, Karolinska Institutet, Stockholm, Sweden; 5 Institute for Global Public Health, University of Manitoba, Winnipeg, Canada; 6 African Population Health and Research Centre, Dakar, Senegal

**Keywords:** Adolescent girls, Sexual and reproductive health, Sub‐saharan Africa, Surveys, Data quality

## Abstract

**Background:**

The annual collection of fertility, marriage, sexual behaviour, and contraceptive use data in the nationally representative rounds of Performance Monitoring and Accountability (PMA) surveys in sub-Saharan Africa may contribute to the periodic monitoring of adolescent sexual and reproductive health (ASRH). However, we need to understand the reliability of these data in monitoring the ASRH indicators. We assessed the internal and external consistencies in ASRH indicators in five countries.

**Methods:**

We included countries with at least three nationally representative rounds of PMA surveys and two recent DHS: Ethiopia, Ghana, Kenya, Nigeria, and Uganda. Our analysis focused on four current status indicators of ASRH among girls 15–19 years: ever had sex, currently married, has given birth or currently pregnant, and currently using modern contraceptives among sexually active unmarried girls. We compared the PMA survey and DHS data and tested for statistical significance and assessed trends over time using Jonckheere-Terpstra test statistic.

**Results:**

PMA and DHS survey methodologies were similar and, where there were differences, these were shown to have minimal impact on the indicator values. The comparison of the data points from PMA and DHS for the same years showed statistically significant differences in 12 of the 20 comparisons, which was most common for sexual behaviour (4/5) and least for contraceptive use (2/5). This is partly due to larger confidence intervals in both surveys. The time trends were consistent between the annual PMA surveys in most instances in Ethiopia, Kenya, and Nigeria but less so for Ghana and Uganda. However, both surveys highlight slow progress in adolescent and reproductive health indicators with major disparities between the countries.

**Conclusions:**

Despite the differences between PMA 2020 surveys and DHS surveys conducted in the same year, and inconsistencies of the PMA survey time series for several indicators in some countries, we found no systematic issues with PMA surveys and consider PMA surveys a valuable data source for the assessment of levels and trends of ASRH beyond contraceptive use and family planning for indicators of fertility, marriage, and sex among adolescent girls in sub-Saharan Africa.

**Supplementary Information:**

The online version contains supplementary material available at 10.1186/s12978-021-01111-0.

## Plain English summary

Adolescent sexual and reproductive health (ASRH) is a priority public health issue in many countries in sub-Saharan Africa, particularly for girls. In most countries in sub-Saharan Africa, monitoring of ASRH relies on national household surveys, predominantly the Demographic and Health Surveys (DHS) which are mostly conducted once every 5 years. This includes contraceptive use as well as sexual activity, marriage, and fertility. The Performance Monitoring and Accountability 2020 (PMA 2020) surveys provide an opportunity to intensify population-based monitoring of trends in key ASRH indicators through annual surveys. While contraceptive use was the primary area of interest, the surveys also collect data on multiple other indicators including fertility, marriage, sexual behaviour. We assessed the extent to which the annual PMA surveys contribute to monitoring of these ASRH indicators by analyzing the annual data and comparing the results with the 5-yearly Demographic and Health Surveys (DHS) in five countries during 2013–2018: Ethiopia, Ghana, Kenya, Nigeria, and Uganda. We assessed the trends and internal consistencies in sex, marriage, pregnancy, and modern contraceptive use among adolescents in five countries. We also assessed the external consistencies by comparing these data with DHS. We focused on four current status indicators of ASRH among girls 15–19 years: ever had sex, currently married, has given birth or currently pregnant, and currently using modern contraceptives among sexually active unmarried girls. In each country, the DHS surveys sample sizes were larger than those of PMA, which could explain the reasons for lager survey design effects and confidence intervals in the PMA surveys as compared to DHS. The PMA and DHS results on adolescent childbearing, child marriage, sexual debut and contraceptive use among unmarried sexually active adolescents (those that had sex within the last 30 days preceding the date of data collection) were different in some countries. However, both surveys’ results highlight slow progress in adolescent and reproductive health indicators with major disparities between the countries. We, therefore, conclude that since adolescent childbearing is an Sustainable Development Goals (SDG) indicator that should receive increased attention as one of the targets and indicators of the SDG, in countries with limited research in adolescent sexual and reproductive data, the PMA surveys are potential data sources for periodic monitoring of adolescent sexual health outcomes and interventions. However, the internal and external inconsistency poses a concern on the reliability of the current surveys in the measurements of ASRH indicators.

## Background

Adolescent sexual and reproductive health (ASRH) is a priority public health issue in many countries in sub-Saharan Africa, particularly for girls [1, 2]. Poor access to modern contraceptives among adolescents in sub-Saharan Africa may lead to adolescent pregnancies and related consequences such as early marriage, school drop-out, and maternal and neonatal morbidity and mortality [1, 2]. Early initiation of sex and low condom use in adolescence is associated with risks of HIV and other sexually transmitted diseases [1–3]. These behavioural patterns have major consequences for adolescent health and development opportunities, especially for girls whose human capital development is undermined [4–7].

In most countries in sub-Saharan Africa, monitoring of ASRH relies on national household surveys, predominantly the Demographic and Health Surveys (DHS) which are mostly conducted once every 5 years [8]. This includes contraceptive use as well as sexual activity, marriage, and fertility. Five-yearly surveys generally should suffice for monitoring marriage, sexual behaviour, and childbearing unless rapid changes are expected. Contraceptive use may change more rapidly during major scale up of interventions and it has been argued that more intensive monitoring may benefit program implementation [9].

The Performance Monitoring and Accountability 2020 (PMA 2020) surveys provide an opportunity to intensify population-based monitoring of trends in key ASRH indicators, as these surveys are conducted frequently. PMA 2020 was primarily created to provide rapid and frequent estimates of modern contraceptive use, demand and supply in priority countries for the global initiative Family Planning 2020 (FP 2020), which aims at a rapid scale-up of access to modern contraception [10]. This monitoring effort in now more than ten countries included household surveys as frequent as once or twice a year. While contraceptive use was the primary area of interest, the surveys also collect data on other indicators including fertility, marriage, sexual behaviour.

The PMA 2020 household surveys present a new approach in terms of its high frequency of implementation, use of resident interviewers  and administering a short face-to-face questionnaire using smartphones or tablets. Details of the PMA survey methodology including data collection processes are published elsewhere [10]. The quality of the PMA survey data on contraceptive use among all women of reproductive ages is considered satisfactory [10]. Also, the use of a limited number of questions on recent births resulted in the only modest underestimation of fertility, largely because of missing multiple births [11]. In this paper, we considered five countries that conducted PMA 2020 and DHS surveys to assess the extent to which PMA 2020 survey data are a useful addition for the tracking of ASRH indicators. We focused on the internal and external consistency of the trends in selected current status indicators of sexual debut, marriage, pregnancy and childbirth, and contraceptive use among adolescent girls, and ascertained trends to assess the value-add of frequent household surveys for monitoring of selected ASRH indicators.

## Methods

We selected countries with at least 3 years of nationally representative rounds of PMA surveys and two recent DHS surveys as of January 2020. Five countries in sub-Saharan Africa met these criteria (Ethiopia, Ghana, Kenya, Nigeria, and Uganda). For Nigeria, data for 2014 and 2015 were not considered as they were not based on a nationally representative sample. We used the IPUMS-PMA database and the DHS archive to download all the datasets [12].

The PMA surveys and DHS both use multistage sampling in the selected clusters. After listing the households within the cluster, a fixed number of households are randomly selected and interviewed. In the PMA surveys, the fixed number of households per cluster is typically 35, ranging from 33 to 44 [13]. Since the PMA surveys revisit the same cluster and households are randomly selected in each round within this cluster, a repeat of respondents who have participated in the previous rounds is possible and this is recorded by the interviewers.

The PMA2020 surveys collect data through female resident enumerators and, unlike the DHS, use the same enumerators (close to 80 %) in subsequent rounds, equipped with a mobile device with uploaded shorter survey questionnaire that is used for face-to-face data collection [14]. Both PMA 2020 surveys and DHS collect information from eligible women aged 15–49 who are either usual members of the household or slept in the household the night before the interview. The sample size in PMA2020 is powered to estimate the modern contraceptive prevalence rate among all women with a margin of error of 3% by sampling strata—typically urban/rural and, in some cases, aggregated administrative regions.

Our analysis focused on four current status indicators among girls 15–19 years:

- Marriage: the proportion of girls who were currently married divided by the total number of girls 15–19 years.
- Ever sexually active: the proportion unmarried adolescent girls (15–19 years) who have ever had sex within the last 30 days preceding the date of data collection.
- Adolescent childbearing: the proportion of girls who were pregnant on the day of the interview or who have ever had a birth among all girls 15–19 years.
- Contraceptive use: the proportion of girls who are currently using modern contraceptives among all unmarried sexually active girls 15–19 years.

There were no major differences between the questionnaire contents that were relevant to these indicators with one exception (Additional file 1: Table S1). Questions about childbearing in the DHS are asked as part of a full birth history, while the PMA2020 is limited to questions about the number of live births and the dates of the most recent births. This may or may not include stillbirths. In addition, the DHS asks separately about live births, and on stillbirths and abortions (Additional file 1: Table S1). We examined the difference.

**Table 1:** PMA surveys and DHS characteristics including the number of clusters surveyed, and the number of respondents 15–19 years (weighted), the percentage of all women 15–49 years who were 15–19 years, and the percentage of girls 15–19 years who were also interviewed in the previous PMA round

Country	Year		All women			
		Number of clusters/EA	Unweighted sample size	% in the previous round	% adolescents interviewed	% in the previous round
Ethiopia	2014 PMA	200	13,241	–	23.3	–
Ethiopia	2015 PMA	200	7631	19.4	24.0	15.9
Ethiopia	2016 PMA	200	7538	22.5	23.1	14.3
Ethiopia	2017 PMA	200	7464	1.3	24.1	1.1
Ethiopia	2018 PMA	200	7546	12.9	22.8	8.0
Ethiopia	2011 DHS	650	16,515	–	23.2	–
Ethiopia	2016 DHS	645	15,683	–	22.3	–
Ghana	2013 PMA	100	3722	–	18.6	–
Ghana	2014 PMA	100	8624	–	19.1	–
Ghana	2015 PMA	100	5254	32.9	20.0	26.5
Ghana	2016 PMA	100	3746	7.7	19.1	8.1
Ghana	2017 PMA	100	4294	16.4	19.7	12.3
Ghana	2008 DHS	412	4916	–	21.1	–
Ghana	2014 DHS	427	9396	–	18.7	–
Kenya	2014 PMA	120	8183	5.9	17.4	4.9
Kenya	2015 PMA	120	9394	18.3	19.8	13.0
Kenya	2016 PMA	120	5973	2.4	21.6	2.5
Kenya	2017 PMA	120	5913	25.8	21.4	16.7
Kenya	2008 DHS	1612	8444	–	20.9	–
Kenya	2014 DHS	400	31,079	–	19.6	–
Nigeria	2016 PMA	37	11,166	8.7	20.2	7.4
Nigeria	2017 PMA	52	13,324	16.7	20.7	13.9
Nigeria	2018 PMA	52	11,215	16.6	20.3	13.4
Nigeria	2013 DHS	904	38,948	–	20.3	–
Nigeria	2018 DHS	1400	41,821	–	20.1	–
Uganda	2014 PMA	110	3762	–	21.0	–
Uganda	2015 PMA	110	7361	22.9	21.3	16.8
Uganda	2016 PMA	110	3816	28.5	20.6	17.9
Uganda	2017 PMA	110	4161	2.1	21.9	2.3
Uganda	2018 PMA	110	4288	15.7	21.8	10.1
Uganda	2011 DHS	712	8674	–	23.4	–
Uganda	2016 DHS	697	18,506	–	23.1	–

PMA2020 conducted two surveys in the same calendar year in all five countries in 2014, and in Kenya in 2015. We combined these two surveys into one because of three reasons (1) the PMA approach of having at least 2 rounds of the survey within the same years was discontinued from 2016; (2) minimal changes within a year in the ASRH indicators are expected; and (3) pooling the datasets increases the PMA sample size for the estimation of annual changes in the ASRH indicators. We assessed the sample characteristics and composition of the PMA and DHS surveys relevant to adolescents.

The analysis was done in STATA 16 and MS Excel. For all indicators, we computed standard errors with their respective 95% confidence for both PMA surveys and DHS. We also computed the survey design effects for PMA surveys and DHS for each indicator. This design effect, referred to DEFF, is the ratio of the actual variance over the variance of a truly randomized sample and is expected to be higher with a smaller number of clusters or with a greater intra-cluster correlation of the indicator of interest [15, 16]. We also assessed the effect of repeated respondents by analysing the data with and without repeat responders.

To assess external consistency, we used a difference of proportions test to assess the statistical significance of the difference between the PMA surveys and DHS for the same country and year for each of the four indicators. To assess internal consistency, we analyzed PMA surveys’ annual trends in the proportion of study indicators over time. We tested for statistical significance of indicators’ trends over time for each of the data source. We applied the same approach in the assessment of the differences between the 1st year of PMA survey and last year of the PMA survey at the time of this study’s data analysis. *Jonter*—a user-written Stata command for testing the trends based on the Jonckheere-Terpstra test [17] was used to test the significance of the trends. For the difference between the data points, we calculate the level of significance based on the data point standard errors. We adjusted for population-weighted numbers in the analysis of indicators with the enumeration areas as the primary sampling unit.

## Results

### Survey characteristics

The number of DHS survey clusters was at least three times higher than those of the annually pooled PMA surveys in each country (Table 1). The number of female respondents 15–19 years was at least two times higher in the DHS than in the PMA surveys (annually pooled), except in Ghana. The proportions of adolescent girls in both surveys, about 20% of all women of reproductive ages, were close in both survey types and varied little over time. We also checked for the response rate for (results not shown) and we found that the response rates for each of the ASRH indicators were at least 95% in both surveys. For instance, sexual debut response rate was at least 95% and 99% the PMA survey in each country for each of the survey round and in the DHS in each country for each of the survey wave, respectively.

The proportion of adolescent girls who had previously participated was variable between countries and PMA surveys (Table 1). It was as high as 26.5% in Ghana in 2015, but less than 10% in nine of the 22 PMA surveys. We examined the effect of repeated respondents in the PMA surveys on the sexual behaviour indicator (ever had sex) by excluding and including the repeated respondents but observed very small absolute changes in the indicator: less than 1% in all surveys except Ethiopia 2015 (1.7%) and Kenya 2017 (2.5%) (Additional file 1: Table S2). The survey design effects were only slightly larger in the PMA surveys than in the DHS. The PMA DEFF relative to DHS DEFF was between 0.9 and 2.0 (Additional file 1: Table S3–S6).

**Table 2:** Summary of findings for consistency between PMA survey and DHS and trends of the respective ASRH indicators by country, based on statistical significance

Indicator	Ethiopia	Kenya	Uganda	Ghana	Nigeria
Consistency PMA survey–DHS					
Sexual debut	=	Yes (H)	Yes (H)	Yes (L)	Yes (H)
Child marriage	=	=	Yes (H)	Yes (H)	Yes (L)
Adolescent birth	=	=	Yes (H)	Yes (L)	Yes (L)
Contraceptive use	No (L)	Yes (L)	No (L)	No (L)	Yes (H)
Trends of the indicator					
Sexual debut	No change	No change	Increase	No change	No change
Child marriage	No change	Decrease	No change	Irregular	Decrease
Adolescent birth	No change	Decrease	Irregular	Irregular	Decrease
Contraceptive use	Decrease (between 2015 and 2018)	Increase	Decrease (between 2015 and 2018)	No change	Increase

H means PMA survey significantly higher than DHS, L means the opposite, =means PMA and DHS estimates are within the same range

Yes means p < 0.05 and no means p > = 0.05

### Adolescent childbearing and pregnancy

**Fig. 1:**
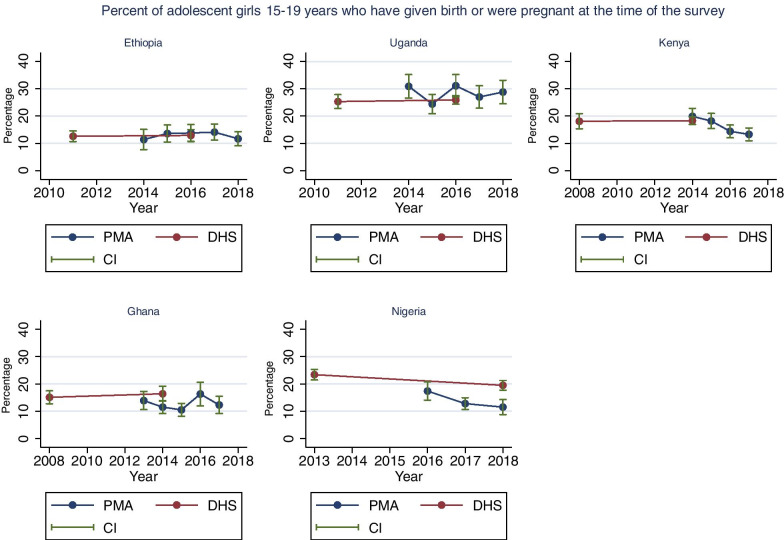
Trends in the percentage of adolescent girls (15-19) who have given birth or were pregant at the time of the surveys. Source: PMA and DHS data recent data as of June 2019

Figure 1 presents the percentage of girls 15–19 years that ever gave birth or were currently pregnant in the PMA surveys and DHS with 95% confidence intervals. The direct comparisons between the PMA survey and DHS conducted in the same year show that there was little difference in Kenya and Ethiopia. The absolute percentage point differences were larger and statistically significant in Uganda (5%, p = 0.02), Nigeria (8%, p = 0.001) and Ghana (5%, p = 0.01).

The trends in the PMA surveys in Ethiopia suggest no change over time (p trend = 0.32). The PMA surveys show an irregular trend in Uganda (between 2014 and 2017) and Ghana (between 2015 and 2017) though none of the PMA surveys differed significantly from the preceding year. Overall, we observed a decrease in the PMA surveys in Nigeria (p trend = 0.06) with a 6% decline observed between 2016 and 2018 (17.4–11.4%, p = 0.007). The Nigeria DHS also showed a slight decline between its last two surveys (23% in 2013 to 20% in 2018) but the inconsistency in levels between the two surveys is large. Kenya is the only country showing a consistent downward trend over time (p trend = 0.02) with a decline from 20% to 2014 to 13% in 2018 (p = 0.007). Details on the PMA and DHS estimates are indicated in Additional file 1: Table S3.

For childbearing, it has to be kept in mind that the PMA surveys and DHS have slightly different questions. The potential size of this difference was assessed in the DHS. The absolute difference between the proportion of adolescent girls who had ever given birth to a live child and the proportion who had a child or stillbirth, or abortion was 0.3% in Kenya DHS 2014, 0.5% in Ethiopia DHS 2015, 1.1% in Nigeria DHS 2018, 1.5% in Uganda DHS 2016 and 2.3% in Ghana DHS 2014. The PMA surveys ask only for births and may have captured some of the stillbirths but would generally be expected to be only slightly lower than DHS (Additional file 1: Table S7).

### Marriage

**Fig. 2:**
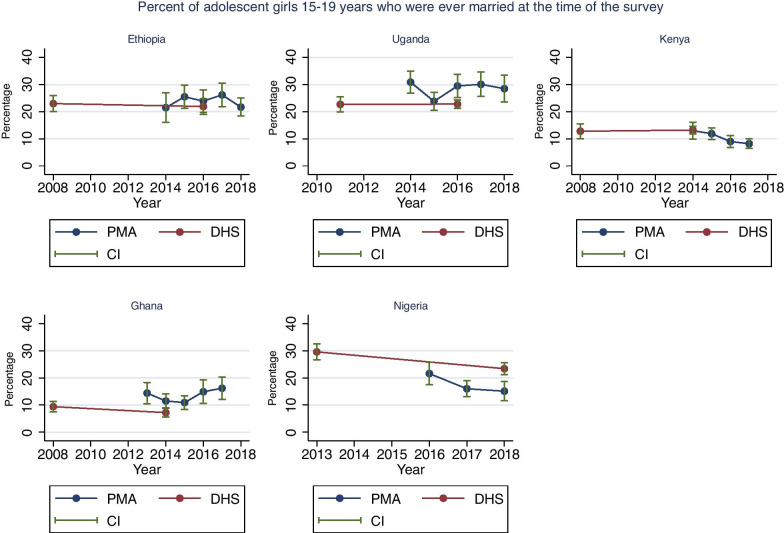
Trends in the percentage of adolescent girls (15-19 years) who were ever married at the time of the surveys. Source: PMA and DHS data recent data as of June 2019

Figure 2 presents the trends in the percentage of adolescents who were ever married at the time of the interview in the PMA surveys and DHS with 95% confidence intervals. The direct comparison between the PMA survey and DHS conducted in the same year shows that there was little difference in Kenya and Ethiopia. The absolute percentage point differences were large and statistically significant in Uganda (7%, p = 0.004), Nigeria (8%, p = 0.001), and Ghana (4%, p = 0.01).

The PMA trends in Ethiopia and Uganda suggest no change over time. In Ghana, the PMA surveys show an irregular trend though none of the surveys differed significantly from the previous year (p trend = 0.33). Overall, a downward trend was observed in Nigeria (p trend = 0.06) with a 7% decline in the PMA surveys between 2016 and 2018 (22% in 2016 to 15% in 2018, p = 0.019). In Kenya, we observed consistent downward trend over time (p trend = 0.02) with a decline from 13 to 8% during 2014–2018 (p = 0.009). Details on the PMA and DHS estimates are indicated in Additional file 1: Table S4.

### Ever had sex

**Fig. 3:**
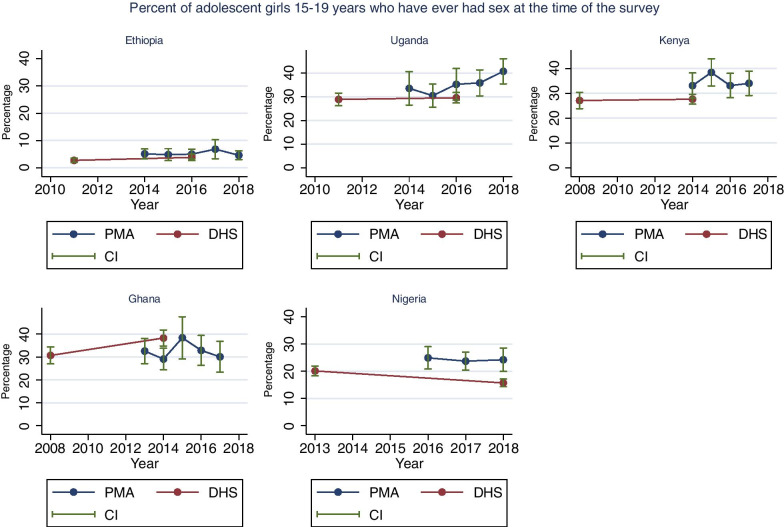
Trends in in the percentange of adolescent girls (15-19 years) who have ever had sex at the time of the surveys. Source: PMA and DHS data recent data as of June 2019

For the percentage of unmarried girls who had ever had sex at the time of the interview the direct comparison between the PMA survey and DHS conducted in the same year shows that there was little difference in Ethiopia (Fig. 3). The absolute percentage point differences were large and statistically significant in Uganda in 2016 (6%, p = 0.11), Kenya in 2014 (6%, p = 0.05), Nigeria in 2018 (9%, p = 0.001), and Ghana in 2014 (9%, p = 0.002).

Overall, we did not observe significant changes in the trend for Kenya (p trend = 0.71), Ethiopia (p trend = 0.62), Nigeria (p trend = 0.60) and Ghana (p trend = 1.00). In Uganda, an upward trend was observed (p trend = 0.02) from 35% to 2014 to 41% in 2018 (p = 0.109). Details on the PMA and DHS estimates are indicated in Additional file 1: Table S5.

### Modern contraceptive use among sexually active adolescents

**Fig. 4:**
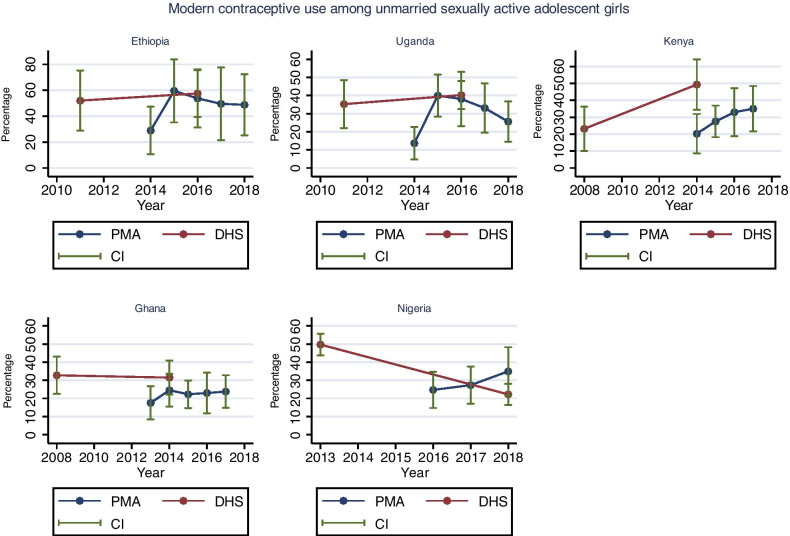
Trends in modern contraceptive use among unmarried sexually active adolescent girls. Source: PMA and DHS data recent data as of June 2019

Figure 4 presents the trends in the percentage of unmarried sexually active adolescents who were using modern contraceptives in the PMA surveys and DHS. The direct comparison between the PMA survey and DHS conducted in the same year shows that there was a large difference in Kenya (20%-PMA versus 49%-DHS, p = 0.001) and, to a lesser extent, in Nigeria (35%-PMA versus 22%-DHS, p = 0.088), and Ghana (25%-PMA versus 31%-DHS, p = 0.298). Differences were small in Uganda and Ethiopia. No significant changes over time were observed in Uganda (p trend = 0.62) and Ethiopia (p trend = 0.62). However, the rates increased by 12% in Uganda between 2014 and 2018 (from 14% to 2014 to 26% in 2018, p = 0.101), and increased by 20% in Ethiopia between 2014 and 2018 (from 29% to 2014 to 49% in 2018, p = 0.193). In Uganda and Ethiopia, the rate first increased between 2014 and 2015 and thereafter steadily declined.

An upward trend was observed in Nigeria (p trend = 0.059) with a 10% increase between 2014 and 2018 (35% in 2014 to 25% in 2018, p = 0.231), Kenya (p trend = 0.02) with a 15% increase between 2014 and 2018 (20% in 2014 to 35% in 2018, p = 0.103), and Ghana (p trend = 0.163) with a 6% increase between 2013 and 2017 (18% in 2013 to 24% in 2018, p = 0.346). Details on the PMA and DHS estimates are indicated in Additional file 1: Table S6.

Table 2 is a summary of findings for consistency between PMA survey and DHS and trends of the respective ASRH indicators by country, based on statistical significance.

## Discussion

The analysis of PMA and DHS surveys in five countries suggests that the PMA surveys are a welcome addition for the monitoring of ASRH indicators, but the large and inconsistency sampling errors between surveys that affect trend analysis are a challenge in several countries. The measure of inconsistency alone may not decisively determine the reliability and validity of the survey; however, using multiple approaches, we compared the data collection tools and results on ASRH indicators of the two surveys.

First, we considered the survey characteristics. PMA surveys used similar survey questions as the DHS for the topics considered here and where there were differences, the impact on the results was small. The re-interviewing of respondents did not influence the results. PMA surveys rely on smaller numbers of clusters and smaller sample size than DHS which resulted in a larger survey design effect and larger sampling errors. The sample compositions in terms of representation of adolescents were similar between PMA surveys and DHS. We also found that the age composition by single years within the 5-year age group varied little and that therefore age standardization had little impact on the results for the indicators. Therefore, we can conclude that the survey compatibility is good and PMA surveys, though smaller in size, provide results that are comparable with DHS. The pooling of survey years for PMA surveys may help reduce the sampling errors.

Second, the comparison of the results for each indicator for the PMA survey and DHS conducted in the same year showed good agreement in Ethiopia for all indicators and in Kenya for all except sexual debut and contraceptive use (Table 2, upper panel). For the other three countries, however, there was a significant difference between the PMA surveys and DHS for all indicators, except family planning in Ghana. The patterns of the differences vary. For instance, in Uganda three of the four indicators were significantly higher than the DHS, while in Ghana the reverse was the case. In general, the PMA survey-based trends were similar to the trend in the last two DHS, but in some countries like Nigeria, the levels were very different. We can conclude that there often are important differences in the results on prevalence for all indicators and that these differences are country specific as they occurred consistently in the three of the five countries and not in Ethiopia and Kenya. This does represent a challenge for the assessment of levels, but the greater consistency for the trends than for levels is important and allows more accurate estimation of levels and trends using all data sources.

Third, to assess the added value of frequent national surveys we reviewed the country trends over time from the PMA surveys and all surveys’ rounds. If the trends were highly irregular, or there is little evidence of changes over time, the added value of annual surveys for monitoring was considered limited. In seven of the 20 country-indicator pairs (five countries and four indicators), there was consistent evidence of changes over time, including child marriage and adolescent fertility in Kenya and Nigeria, and sexual debut and family planning use in Uganda. Even though high-frequency surveys are more useful for indicators that can change rapidly (e.g., coverage of interventions that receive large investments) than for indicators of behaviour such as marriage, sex and childbearing in adolescence which tend to change more gradually, we did not find consistent evidence to support this. The large sampling errors in the annual surveys are a likely contributor.

Our findings are consistent with earlier assessments of the data quality and added value of PMA surveys on contraceptive use [10] and fertility [11]. The surveys provide a wealth of information on, in this case, ASRH indicators which allow more frequent tracking, especially when used in combination with DHS results. The surveys are, however, smaller in terms of numbers of clusters and sample size and disaggregation by subnational areas or wealth quintiles including other stratifiers could be problematic. The added value for monitoring indicators of sexual behaviour, marriage and childbearing lies primarily in adding to the volume of data generated in countries through multiple survey platforms. Pooling data sets will allow for greater disaggregation. Given the relatively slow change of these culturally engrained ASRH behaviours related to sex, marriage, and childbearing, it appears that annual monitoring would add only limited value, also given the sampling errors and observed differences between surveys. This, however, would be different for indicators such as contraceptive use which could change more rapidly. The utility of annual surveys would then be determined by expected annual changes related to the level of program effort.

Despite the internal and external inconsistencies, our analyses confirm that ASRH remains a critical issue for girls in the five countries in sub-Saharan Africa. Based on the PMA annual estimates, sexual debut in adolescence is common and not decreasing, with 30–40% of 15–19-year-old single girls reporting to have started sex in four of the five countries. while the current use of modern contraceptives among unmarried adolescent girls remained low and puts girls at risk of pregnancy and sexually transmitted infections. Low use of modern contraceptives among unmarried adolescent girls, especially condoms, has been raised as a major issue for ASRH programs [18]. Furthermore, child marriage and adolescent childbearing continued to be common in all countries affecting 10–25% and 10–30% of girls respectively in the most recent PMA surveys, and only in Kenya and perhaps Nigeria, there was evidence of a decline of both indicators.

There are striking differences between the five countries. At the one end, Uganda stands out with close to 30% of girls 15–19 who are pregnant or have begun childbearing, compared to only 10% in the other four countries according to the most recent PMA survey. Also, the proportion married and early initiation of sex is greater in Uganda than in the other countries. Ethiopia is at the other end, characterized by low sexual activity among unmarried adolescent girls, low levels of adolescent childbearing and high levels of child marriage. The contraceptive use among unmarried sexually active adolescents close to 50% in Ethiopia compared to 25% in Ghana and Uganda, and 35% in Kenya and Nigeria. The Kenya PMA surveys provide the greatest evidence of a decline in ASRH indicators of sex—marriage and childbearing. Nigeria shows an improvement in several ASRH indicators, while Ghana’s trend data, the most irregular of the five countries, present no evidence of positive changes. Adolescent childbearing is an SDG indicator which should receive increased attention as one of the targets (and indicators) of the SDGs but the evidence of positive changes is modest.

## Limitations

Our analysis is based on data from five countries and may not capture the potentially larger variation between surveys in other country situations with different patterns of ASRH-related behaviours. PMA 2020 surveys have now expanded to 11 countries which open the possibility to conduct further comparative analyses provided DHS or other surveys are available. The five countries used for our analysis also present extremes of a wide spectrum of ASRH patterns in sub-Saharan Africa, as shown in a paper by Melesse et al. in the Supplement. The observed differences between the PMA surveys and DHS results in some of the countries are not due to sampling errors alone but also survey implementation, design issues or other issues that were beyond the scope of this study.

DHS and PMA survey data on ASRH are based on recall of self-reported behaviour collected through face–face interviews. Such data could lead to non-sampling biases and thus affect the survey results, and such could perhaps explain the differences between the DHS and PMA estimates. Despite the high response rates, we know that the accurate reporting of premarital sex, premarital pregnancies and marriage depend on context, culture, confidentiality, legal and moral norms, as well as survey characteristics such as interviewer characteristics and training, length and structure of the questionnaire [19, 20]. Studies have indicated that the behaviours that are in contrast to widespread of social norms and regulations may be under-reported [21, 22] particularly for the interviews that are conducted face-to-face or those that may reveal the respondents’ identity [23, 24]. A PMA report on the methodological issues related to the reporting of the sensitive behaviours indicated that there were some inconsistencies in the reporting of the questions such as the number of births [14], which may affect the adolescent pregnancy or birth estimates of such indicators. Additionally, marriage and union formation are often not single events—and because of the legal, cultural, and regional attached to such events, such may lead to underestimation if other aspects like cohabiting or living with a partner like you are married are not captured. Such factors may generate socially desirable responses rather than accurate information [25, 26] and thus leading to underestimation. The misreporting arising from such could be more prevalent among adolescents who are not yet comfortable with their sexuality [25] because of the societal perceptions and legalities around early sex. The issue of greatest concern is the underreporting of events. A study that compared the same PMA survey and telephone interviews’ respondents on the estimation of modern contraceptive prevalence indicated significant differences with the telephone interviews indicating higher estimated [27]. In addition to ensuring privacy and confidentiality during interview, other innovation approaches such as computer assisted interviews and confidential voting interviews [23, 28–30] that have been applied to reduce social desirability bias should be considered for such sensitive questions.

In general, our study did not result in the identification of possible structural differences in the non-sampling biases between DHS and PMA surveys. In the three countries with significant differences between the two surveys the direction of the difference varied by indicator and by country, but no clear pattern emerged.

## Conclusions

This study contributes to the literature on the reliability of the available data sources in the measurement of ASRH indicators for girls and how these data could be used to monitor trends. The internal inconsistencies in the ASRH changes in the PMA surveys and external inconsistencies in some of the countries presents a concern on the reliability of the available data in estimating ASRH indicators. Further, the small samples may also make it impossible for the examination of the distribution of ASRH indicators across stratifiers e.g., socio-economic characteristics and further data pooling should be considered to overcome this challenge. Despite the differences between PMA 2020 surveys and DHS surveys conducted in the same year, and inconsistencies of the PMA survey time series for several indicators in some countries, we found no systematic issues with PMA surveys and consider PMA surveys a valuable data source for the assessment of levels and trends of ASRH beyond contraceptive use and family planning for indicators of fertility, marriage, and sex among adolescent girls in sub-Saharan Africa.

## Supplementary Information

**Additional file 1: Table S1.** Comparing birth, sex and marriage questions asked in both PMA and DHS surveys.** Table S2.** Trends in adolescents who have ever had sex using all rounds PMA2020 data for assessing the effect of repeated respondent participation in subsequent surveys.** Table S3.** PMA and recent DHS weighted estimates for adolescents who have ever been pregnant with their respective standard errors and design effect.** Table S4.** PMA and recent DHS weighted estimates for adolescents who have ever been married with their respective standard errors and design effect.** Table S5.** PMA and recent DHS weighted estimates for unmarried adolescents who have ever had sex with their respective standard errors and design effect.** Table S6.** PMA and recent DHS weighted estimates for unmarried sexually active adolescents who use contraceptives with their respective standard errors and design effect.** Table S7.** Recent DHS estimates for the adolescents who were ever pregnant.

## Data Availability

Datasets are publicly available at https://dhsprogram.com/ and https://www.pmadata.org/. The PMA2020 data considered in this study were all rounds collected between 2013 and 2018 that were publicly available as of January 2020. The DHS data considered in this study were the two most recent surveys that were publicly available as of January 2020.

## Abbreviations

ASRH: Adolescent sexual and reproductive health
DEFF: Design effect
DHS: Demographic and Health Surveys
FP: Family planning
HIV: Human immunodeficiency virus
PMA: Performance Monitoring and Accountability
