# Supplementary material for: Examining the recent trends in adolescent sexual and reproductive health in five countries of sub‐Saharan Africa based on PMA and DHS household surveys
Source: Reprod Health. 2021 Jun 17;18(Suppl 1):121. doi: 10.1186/s12978-021-01111-0 (PMC8210352; doi:10.1186/s12978-021-01111-0)
Supplement: Supplementary file 1 — Additional file 1: Table S1. Comparing birth, sex and marriage questions asked in both PMA and DHS surveys. Table S2. Trends in adolescents who have ever had sex using all rounds PMA2020 data for assessing the effect of repeated respondent participation in subsequent surveys. Table S3. PMA and recent DHS weighted estimates for adolescents who have ever been pregnant with their respective standard errors and design effect. Table S4. PMA and recent DHS weighted estimates for adolescents who have ever been married with their respective standard errors and design effect. Table S5. PMA and recent DHS weighted estimates for unmarried adolescents who have ever had sex with their respective standard errors and design effect. Table S6. PMA and recent DHS weighted estimates for unmarried sexually active adolescents who use contraceptives with their respective standard errors and design effect. Table S7. Recent DHS estimates for the adolescents who were ever pregnant. [file 12978_2021_1111_MOESM1_ESM.docx]

**Additional file**

**Table S1: Comparing the birth, sex and marriage questions asked in both PMA and DHS surveys**

|  | **PMA2020** | **DHS** |
| --- | --- | --- |
| **Pregnancy and birth history** |  |  |
| Are you pregnant now? | Yes | Yes |
| Have you ever had a pregnancy that miscarried, was aborted, or ended in a stillbirth? | No | Yes |
| In what month and year did the preceding such pregnancy end? | No | Yes |
| How many months pregnant were you when that pregnancy ended? | No | Yes |
| How many times have you given birth? | Yes | Yes |
| Have you ever given birth? | Yes | Yes |
| **Sex debut history** |  |  |
| How old were you when you first had sexual intercourse? | Yes | Yes |
| **Marriage history** |  |  |
| Are you currently married or living together with a man as if married? | Yes | Yes |
| Have you been married or lived with a man only once or more than once? | Yes | Yes |
| In what month and year did you start living with your FIRST husband/partner? | Yes | Yes |

**Table S2: Trends in adolescents who have ever had sex using all rounds PMA2020 data for assessing the effect of repeated respondent participation in subsequent surveys**

|  |  | Excluding those who previously participated | | All participants included | |
| --- | --- | --- | --- | --- | --- |
| Country | Year | Weighted Percent | 95% CI | Weighted percent | 95% CI |
| Ethiopia | 2014 | 24.8 | 23.2-26.4 | 24.8 | 23.2-26.4 |
| Ethiopia | 2015 | 29.8 | 27.5-32.1 | 28.1 | 26.1-30.2 |
| Ethiopia | 2016 | 26.3 | 24.0-28.5 | 26.8 | 24.7-28.8 |
| Ethiopia | 2017 | 30.5 | 28.3-32.7 | 30.6 | 28.4-32.7 |
| Ethiopia | 2018 | 24.4 | 22.3-26.5 | 24.6 | 22.5-26.6 |
| Ghana | 2013 | 39.2 | 35.9-42.5 | 39.2 | 35.9-42.5 |
| Ghana | 2014 | 35.9 | 33.5-38.3 | 35.9 | 33.5-38.3 |
| Ghana | 2015 | 39.9 | 36.5-43.2 | 40.6 | 37.6-43.5 |
| Ghana | 2016 | 38.4 | 34.7-42.2 | 39.2 | 35.6-42.8 |
| Ghana | 2017 | 39.4 | 35.8-43.0 | 40.2 | 36.8-43.5 |
| Kenya | 2014 | 38.9 | 36.2-41.6 | 38.6 | 35.9-41.2 |
| Kenya | 2015 | 44.4 | 41.9-47.0 | 43.5 | 41.1-45.9 |
| Kenya | 2016 | 37.7 | 35.1-40.4 | 37.8 | 35.1-40.4 |
| Kenya | 2017 | 35.9 | 33.0-38.8 | 38.7 | 36.0-41.4 |
| Nigeria | 2016 | 39.3 | 37.1-41.4 | 39 | 36.9-41.1 |
| Nigeria | 2017 | 34.2 | 32.2-36.1 | 34.4 | 32.6-36.3 |
| Nigeria | 2018 | 34.7 | 32.5-36.9 | 34.1 | 32.1-36.1 |
| Uganda | 2014 | 49.8 | 46.3-53.4 | 49.8 | 46.3-53.4 |
| Uganda | 2015 | 44.8 | 42.1-47.5 | 45.5 | 43.0-48.0 |
| Uganda | 2016 | 52.9 | 49.1-56.8 | 53 | 49.5-56.5 |
| Uganda | 2017 | 54.4 | 51.1-57.8 | 54.3 | 51.0-57.6 |
| Uganda | 2018 | 56.4 | 53.0-59.7 | 56.9 | 53.7-60.1 |
| **Note:** *The estimates include both married and unmarried adolescents* | | | | | |

**Table S3: PMA and recent DHS weighted estimates for adolescents who have ever been pregnant with their respective standard errors and design effect**

|  |  | **Currently or ever pregnant** | | | | | | | |  |
| --- | --- | --- | --- | --- | --- | --- | --- | --- | --- | --- |
|  |  | **PMA** | | | **DHS** | | | **Difference** | |  |
| Country | Year | Weighted proportion (P1) | SE-PMA | DEFF-PMA | Weighted proportion(P2) | SE-DHS | DEFF-DHS | Proportion (P2-P1) | SE | DEFF-PMA relative DEFF-DHS |
| Ethiopia | 2011 | *-* | *-* | *-* | 0.126 | 0.01 | 3.8 | 0.01 | 0.021 | 2.7 |
| Ethiopia | 2014 | 0.114 | 0.019 | 10.3 | *-* | *-* | *-* | *-* | *-* | *-* |
| Ethiopia | 2015 | 0.136 | 0.016 | 4.1 | *-* | *-* | *-* | *-* | *-* | *-* |
| Ethiopia | 2016 | 0.138 | 0.016 | 3.9 | 0.129 | 0.011 | 4 | -0.01 | 0.019 | 1.0 |
| Ethiopia | 2017 | 0.141 | 0.015 | 3.6 | *-* | *-* | *-* | *-* | *-* | *-* |
| Ethiopia | 2018 | 0.117 | 0.013 | 3 | *-* | *-* | *-* | *-* | *-* | *-* |
| Ghana | 2008 | *-* | *-* | *-* | 0.151 | 0.012 | 1.2 | 0.01 | 0.021 | 1.7 |
| Ghana | 2013 | 0.139 | 0.017 | 2 |  |  |  | *-* | *-* | *-* |
| Ghana | 2014 | 0.115 | 0.012 | 2.1 | 0.164 | 0.014 | 2.3 | 0.05 | 0.018 | 0.9 |
| Ghana | 2015 | 0.105 | 0.012 | 1.6 | *-* | *-* | *-* | *-* | *-* | *-* |
| Ghana | 2016 | 0.163 | 0.022 | 2.5 | *-* | *-* | *-* | *-* | *-* | *-* |
| Ghana | 2017 | 0.123 | 0.016 | 2 | *-* | *-* | *-* | *-* | *-* | *-* |
| Kenya | 2008 | *-* | *-* | *-* | 0.181 | 0.014 | 2.3 | -0.02 | 0.021 | 0.9 |
| Kenya | 2014 | 0.199 | 0.015 | 2.1 | 0.183 | 0.007 | 2.1 | -0.02 | 0.017 | 1.0 |
| Kenya | 2015 | 0.182 | 0.014 | 2.4 | *-* | *-* | *-* | *-* | *-* | *-* |
| Kenya | 2016 | 0.144 | 0.012 | 1.5 | *-* | *-* | *-* | *-* | *-* | *-* |
| Kenya | 2017 | 0.133 | 0.012 | 1.7 | *-* | *-* | *-* | *-* | *-* | *-* |
| Nigeria | 2013 | *-* | *-* | *-* | 0.234 | 0.01 | 4.7 | 0.06 | 0.020 | 1.0 |
| Nigeria | 2016 | 0.174 | 0.017 | 4.5 | *-* | *-* | *-* | *-* | *-* | *-* |
| Nigeria | 2017 | 0.128 | 0.011 | 2.9 | *-* | *-* | *-* | *-* | *-* | *-* |
| Nigeria | 2018 | 0.115 | 0.014 | 4 | 0.195 | 0.009 | 4 | 0.08 | 0.017 | 1.0 |
| Uganda | 2011 | *-* | *-* | *-* | 0.253 | 0.013 | 1.9 | -0.06 | 0.026 | 0.9 |
| Uganda | 2014 | 0.309 | 0.022 | 1.7 | *-* | *-* | *-* | *-* | *-* | *-* |
| Uganda | 2015 | 0.244 | 0.018 | 2.9 | *-* | *-* | *-* | *-* | *-* | *-* |
| Uganda | 2016 | 0.311 | 0.021 | 1.6 | 0.259 | 0.008 | 1.6 | -0.052 | 0.022 | 1.0 |
| Uganda | 2017 | 0.27 | 0.021 | 2 | *-* | *-* | *-* | *-* | *-* | *-* |
| Uganda | 2018 | 0.288 | 0.022 | 2.2 | *-* | *-* | *-* | *-* | *-* | *-* |

**Table S4: PMA *and recent DHS* weighted estimates *for adolescents who have ever been married with* their respective standard errors and design effect**

|  |  | **Currently married** | | | | | | | | |
| --- | --- | --- | --- | --- | --- | --- | --- | --- | --- | --- |
|  |  | **PMA** | | | **DHS** | | | **Difference** | |  |
| Country | Year | Weighted proportion (P1) | SE-PMA | DEFF-PMA | Weighted proportion(P2) | SE-DHS | DEFF-DHS | Proportion (P2-P1) | SE | DEFF-PMA relative DEFF-DHS |
| Ethiopia | 2008 | *-* | *-* | *-* | 0.23 | 0.015 | 5.3 | -0.02 | 0.032 | 2.5 |
| Ethiopia | 2014 | 0.215 | 0.028 | 13.4 | *-* | *-* | *-* | *-* | *-* | *-* |
| Ethiopia | 2015 | 0.255 | 0.022 | 4.5 | *-* | *-* | *-* | *-* | *-* | *-* |
| Ethiopia | 2016 | 0.239 | 0.021 | 4.4 | 0.219 | 0.015 | 4.7 | 0.02 | 0.026 | 0.9 |
| Ethiopia | 2017 | 0.262 | 0.022 | 4.6 | *-* | *-* | *-* | *-* | *-* | *-* |
| Ethiopia | 2018 | 0.217 | 0.017 | 3.2 | *-* | *-* | *-* | *-* | *-* | *-* |
| Ghana | 2008 | *-* | *-* | *-* | 0.094 | 0.01 | 1.3 | 0.05 | 0.022 | 2.2 |
| Ghana | 2013 | 0.144 | 0.02 | 2.8 | *-* | *-* | *-* | *-* | *-* | *-* |
| Ghana | 2014 | 0.115 | 0.013 | 2.5 | 0.072 | 0.008 | 1.4 | 0.04 | 0.015 | 1.8 |
| Ghana | 2015 | 0.109 | 0.013 | 2 | *-* | *-* | *-* | *-* | *-* | *-* |
| Ghana | 2016 | 0.149 | 0.022 | 2.8 | *-* | *-* | *-* | *-* | *-* | *-* |
| Ghana | 2017 | 0.162 | 0.021 | 2.9 | *-* | *-* | *-* | *-* | *-* | *-* |
| Kenya | 2008 | *-* | *-* | *-* | 0.128 | 0.014 | 2.9 | 0.00 | 0.021 | 1.0 |
| Kenya | 2014 | 0.13 | 0.016 | 2.9 | 0.132 | 0.007 | 2.3 | 0.00 | 0.017 | 1.3 |
| Kenya | 2015 | 0.119 | 0.011 | 2.1 | *-* | *-* | *-* | *-* | *-* | *-* |
| Kenya | 2016 | 0.09 | 0.011 | 1.8 | *-* | *-* | *-* | *-* | *-* | *-* |
| Kenya | 2017 | 0.082 | 0.009 | 1.5 | *-* | *-* | *-* | *-* | *-* | *-* |
| Nigeria | 2013 | *-* | *-* | *-* | 0.296 | 0.015 | 8.3 | -0.08 | 0.026 | 0.7 |
| Nigeria | 2016 | 0.216 | 0.021 | 5.5 | *-* | *-* | *-* | *-* | *-* | *-* |
| Nigeria | 2017 | 0.16 | 0.015 | 4.2 | *-* | *-* | *-* | *-* | *-* | *-* |
| Nigeria | 2018 | 0.151 | 0.018 | 5.5 | 0.234 | 0.011 | 5.4 | 0.08 | 0.021 | 1.0 |
| Uganda | 2011 | *-* | *-* | *-* | 0.227 | 0.014 | 2.3 | 0.08 | 0.025 | 0.7 |
| Uganda | 2014 | 0.309 | 0.021 | 1.6 | *-* | *-* | *-* | *-* | *-* | *-* |
| Uganda | 2015 | 0.238 | 0.017 | 2.6 | *-* | *-* | *-* | *-* | *-* | *-* |
| Uganda | 2016 | 0.295 | 0.022 | 1.9 | 0.228 | 0.008 | 1.8 | 0.07 | 0.023 | 1.1 |
| Uganda | 2017 | 0.301 | 0.023 | 2.3 | *-* | *-* | *-* | *-* | *-* | *-* |
| Uganda | 2018 | 0.285 | 0.025 | 2.9 | *-* | *-* | *-* | *-* | *-* | *-* |

**Table S5: PMA *and recent DHS* weighted estimates *for unmarried adolescents who have ever had sex* with their respective standard errors and design effect**

|  |  | **Ever sex among unmarried** | | | | | | | | |
| --- | --- | --- | --- | --- | --- | --- | --- | --- | --- | --- |
|  |  | **PMA** | | | **DHS** | | | **Difference** | |  |
| Country | Year | Weighted proportion (P1) | SE-PMA | DEFF-PMA | Weighted proportion(P2) | SE-DHS | DEFF-DHS | Proportion (P2-P1) | SE | DEFF-PMA relative DEFF-DHS |
| Ethiopia | 2011 | *-* | *-* | *-* | 0.027 | 0.004 | 2.4 | 0.02 | 0.010 | 1.5 |
| Ethiopia | 2014 | 0.051 | 0.009 | 3.7 | *-* | *-* | *-* | *-* | *-* | *-* |
| Ethiopia | 2015 | 0.048 | 0.011 | 3.4 | *-* | *-* | *-* | *-* | *-* | *-* |
| Ethiopia | 2016 | 0.05 | 0.009 | 2.2 | 0.038 | 0.006 | 2.3 | 0.01 | 0.011 | 1.0 |
| Ethiopia | 2017 | 0.068 | 0.018 | 6.8 | *-* | *-* | *-* | *-* | *-* | *-* |
| Ethiopia | 2018 | 0.046 | 0.008 | 2.1 | *-* | *-* | *-* | *-* | *-* | *-* |
| Ghana | 2008 | *-* | *-* | *-* | 0.307 | 0.019 | 1.6 | 0.02 | 0.034 | 1.5 |
| Ghana | 2013 | 0.326 | 0.028 | 2.4 |  |  |  | *-* | *-* | *-* |
| Ghana | 2014 | 0.291 | 0.024 | 3.8 | 0.383 | 0.018 | 2 | -0.09 | 0.030 | 1.9 |
| Ghana | 2015 | 0.384 | 0.047 | 8.8 | *-* | *-* | *-* | *-* | *-* | *-* |
| Ghana | 2016 | 0.329 | 0.033 | 3.1 | *-* | *-* | *-* | *-* | *-* | *-* |
| Ghana | 2017 | 0.301 | 0.034 | 4 | *-* | *-* | *-* | *-* | *-* | *-* |
| Kenya | 2008 | *-* | *-* | *-* | 0.271 | 0.017 | 2.3 | 0.06 | 0.031 | 1.4 |
| Kenya | 2014 | 0.331 | 0.026 | 3.3 | 0.276 | 0.01 | 2.8 | -0.06 | 0.028 | 1.2 |
| Kenya | 2015 | 0.384 | 0.028 | 4.9 | *-* | *-* | *-* | *-* | *-* | *-* |
| Kenya | 2016 | 0.331 | 0.025 | 3.5 | *-* | *-* | *-* | *-* | *-* | *-* |
| Kenya | 2017 | 0.34 | 0.025 | 3.2 | *-* | *-* | *-* | *-* | *-* | *-* |
| Nigeria | 2013 | *-* | *-* | *-* | 0.201 | 0.009 | 3 | 0.05 | 0.023 | 1.2 |
| Nigeria | 2016 | 0.249 | 0.021 | 3.7 | *-* | *-* | *-* | *-* | *-* | *-* |
| Nigeria | 2017 | 0.237 | 0.017 | 3.5 | *-* | *-* | *-* | *-* | *-* | *-* |
| Nigeria | 2018 | 0.242 | 0.022 | 4.6 | 0.157 | 0.007 | 2.3 | 0.09 | 0.023 | 2.0 |
| Uganda | 2011 | *-* | *-* | *-* | 0.289 | 0.014 | 1.6 | 0.05 | 0.039 | 1.8 |
| Uganda | 2014 | 0.336 | 0.036 | 2.9 | *-* | *-* | *-* | *-* | *-* | *-* |
| Uganda | 2015 | 0.305 | 0.025 | 3.7 | *-* | *-* | *-* | *-* | *-* | *-* |
| Uganda | 2016 | 0.353 | 0.034 | 2.9 | 0.296 | 0.011 | 1.9 | -0.06 | 0.036 | 1.5 |
| Uganda | 2017 | 0.359 | 0.028 | 2.1 | *-* | *-* | *-* | *-* | *-* | *-* |
| Uganda | 2018 | 0.408 | 0.027 | 2 | *-* | *-* | *-* | *-* | *-* | *-* |

**Table S6: PMA *and recent DHS* weighted estimates *for unmarried sexually active adolescents who use contraceptives* with their respective standard errors and design effect**

|  |  | **Contraceptive use among all sexually active adolescents** | | | |  |  |  |  |  |
| --- | --- | --- | --- | --- | --- | --- | --- | --- | --- | --- |
|  |  | **PMA** | | | **DHS** | | | **Difference** | | |
| Country | Year | Weighted proportion (P1) | SE-PMA | DEFF-PMA | Weighted proportion(P2) | SE-DHS | DEFF-DHS | Proportion (P2-P1) | SE | DEFF-PMA relative DEFF-DHS |
| Ethiopia | 2011 |  |  |  | 0.52 | 0.12 | 2.08 | -0.23 | 0.151 | 1.224 |
| Ethiopia | 2014 | 0.29 | 0.09 | 2.54 |  |  |  | *-* | *-* | *-* |
| Ethiopia | 2015 | 0.60 | 0.12 | 2.24 |  |  |  | *-* | *-* | *-* |
| Ethiopia | 2016 | 0.54 | 0.11 | 2.23 | 0.58 | 0.09 | 1.87 | -0.04 | 0.147 | 1.193 |
| Ethiopia | 2017 | 0.50 | 0.14 | 3.40 |  |  |  | - | - | *-* |
| Ethiopia | 2018 | 0.49 | 0.12 | 2.20 |  |  |  |  |  | *-* |
| Ghana | 2008 |  |  |  | 0.33 | 0.05 | 1.19 | -0.15 | 0.070 | 1.212 |
| Ghana | 2013 | 0.18 | 0.05 | 1.44 |  |  |  | *-* | *-* | *-* |
| Ghana | 2014 | 0.25 | 0.05 | 1.97 | 0.31 | 0.05 | 2.02 | -0.07 | 0.067 | 0.976 |
| Ghana | 2015 | 0.22 | 0.04 | 1.76 |  |  |  | *-* | *-* | *-* |
| Ghana | 2016 | 0.23 | 0.06 | 2.07 |  |  |  | *-* | *-* | *-* |
| Ghana | 2017 | 0.24 | 0.05 | 1.25 |  |  |  | *-* | *-* | *-* |
| Kenya | 2008 |  |  |  | 0.23 | 0.07 | 1.93 | -0.03 | 0.089 | 0.986 |
| Kenya | 2014 | 0.20 | 0.06 | 1.90 | 0.49 | 0.08 | 1.99 | -0.29 | 0.096 | 0.953 |
| Kenya | 2015 | 0.28 | 0.05 | 2.35 |  |  |  | - | - | *-* |
| Kenya | 2016 | 0.33 | 0.07 | 3.00 |  |  |  | - | - | *-* |
| Kenya | 2017 | 0.35 | 0.07 | 2.91 |  |  |  | - | - | *-* |
| Nigeria | 2013 |  |  |  | 0.50 | 0.03 | 1.45 | -0.25 | 0.059 | 1.724 |
| Nigeria | 2016 | 0.25 | 0.05 | 2.49 |  |  |  | - | - | *-* |
| Nigeria | 2017 | 0.27 | 0.05 | 3.20 |  |  |  | - | - | *-* |
| Nigeria | 2018 | 0.35 | 0.07 | 3.54 | 0.22 | 0.03 | 1.67 | 0.13 | 0.074 | 2.123 |
| Uganda | 2011 |  |  |  | 0.35 | 0.07 | 1.74 | -0.22 | 0.082 | 0.651 |
| Uganda | 2014 | 0.14 | 0.05 | 1.13 |  |  |  | - | - | *-* |
| Uganda | 2015 | 0.40 | 0.06 | 1.79 |  |  |  | - | - | *-* |
| Uganda | 2016 | 0.38 | 0.08 | 1.83 | 0.40 | 0.04 | 1.53 | -0.02 | 0.086 | 1.190 |
| Uganda | 2017 | 0.33 | 0.07 | 1.67 |  |  |  | - | - | *-* |
| Uganda | 2018 | 0.26 | 0.06 | 1.50 |  |  |  |  |  | *-* |

**Table S7: Recent DHS estimates for the adolescents who were ever pregnant**

|  |  | **Birth excluding stillbirth and abortions** | | **Births including stillbirths and abortions** | | **All pregnant and birth events** | |
| --- | --- | --- | --- | --- | --- | --- | --- |
| *Country* | *year* | *Weighted percent* | *95% CI* | *Weighted percent* | *95% CI* | *Weighted percent* | *95% CI* |
| Nigeria | 2018 | 14.4 | 13.1-15.8 | 15.5 | 14.2-17.0 | 19.5 | 17.9-21.3 |
| Ghana | 2014 | 11.3 | 9.8-12.9 | 13.6 | 11.9-15.3 | 16.4 | 14.6-18.3 |
| Kenya | 2014 | 14.7 | 13.8-15.6 | 15 | 14.1-15.9 | 18.3 | 17.3-19.3 |
| Ethiopia | 2016 | 10.1 | 9.0-11.1 | 10.6 | 9.6-11.7 | 12.9 | 11.8-14.0 |
| Uganda | 2016 | 19.4 | 18.2-20.6 | 20.9 | 19.7-22.1 | 25.9 | 24.6-27.2 |
